# Home inotrope therapy in chronic stimulant-induced cardiomyopathy: a case series

**DOI:** 10.1093/ehjcr/ytae406

**Published:** 2024-08-12

**Authors:** Max Joseph, Sejal Batra, Wali Kamran, Kelsey Barrett, Barbara Ebert, Ahmed Nassar, Timothy Misselbeck, Nael Hawwa

**Affiliations:** Lehigh Valley Heart and Vascular Institute, Lehigh Valley Health Network, 1250 S Cedar Crest Blvd #300, Allentown, PA 18103, USA; Lehigh Valley Heart and Vascular Institute, Lehigh Valley Health Network, 1250 S Cedar Crest Blvd #300, Allentown, PA 18103, USA; Lehigh Valley Heart and Vascular Institute, Lehigh Valley Health Network, 1250 S Cedar Crest Blvd #300, Allentown, PA 18103, USA; Lehigh Valley Heart and Vascular Institute, Lehigh Valley Health Network, 1250 S Cedar Crest Blvd #300, Allentown, PA 18103, USA; Lehigh Valley Heart and Vascular Institute, Lehigh Valley Health Network, 1250 S Cedar Crest Blvd #300, Allentown, PA 18103, USA; Lehigh Valley Heart and Vascular Institute, Lehigh Valley Health Network, 1250 S Cedar Crest Blvd #300, Allentown, PA 18103, USA; Lehigh Valley Heart and Vascular Institute, Lehigh Valley Health Network, 1250 S Cedar Crest Blvd #300, Allentown, PA 18103, USA; Lehigh Valley Heart and Vascular Institute, Lehigh Valley Health Network, 1250 S Cedar Crest Blvd #300, Allentown, PA 18103, USA

**Keywords:** Methamphetamine, Cardiogenic shock, Home inotrope, Case report

## Abstract

**Background:**

Patients with chronic stimulant-induced cardiomyopathy presenting with cardiogenic shock can be stabilized with conventional measures. However, their management post-stabilization has not been well described and poses unique challenges: (i) less chance of myocardial recovery compared to acute stimulant-induced cardiomyopathy, (ii) psychosocial barriers to left ventricular assist device (LVAD) and heart transplantation, and (iii) concern for use of peripherally inserted central catheter for home inotrope in those with a history of substance abuse.

**Case summary:**

Three patients with chronic stimulant-induced cardiomyopathy were admitted with cardiogenic shock progressing to Society for Cardiovascular Angiography & Interventions stage D or E. They were stabilized with inotrope and/or biventricular mechanical circulatory support. Long-term home inotrope was used as either a bridge to LVAD, reverse remodelling, or stabilization.

**Discussion:**

Home inotrope should be viewed as an option in chronic stimulant-induced cardiomyopathy on a case-by-case basis. It can buy time to allow for myocardial stabilization or recovery through goal-directed medical therapy and stimulant cessation. It can also serve as a ‘psychosocial stress test’ for future consideration of advanced heart failure therapies.

**Learning points**

- Home inotrope is an option in stimulant-induced cardiomyopathy on a case-by-case basis.
- The ability to manage a peripherally inserted central catheter and home inotrope can provide valuable psychosocial information during left ventricular assist device and heart transplant evaluation.
- The indirect inotropic effect of methamphetamine is an additional source of drug dependence in patients with severe cardiomyopathy.

## Introduction

Stimulant use has been reported to cause dilated cardiomyopathy (DCM).^1^ Patients with chronic stimulant-induced DCM can present with cardiogenic shock. Following their stabilization, the risk/benefit of long-term home inotrope and trajectory of these patients are not well described. We present three such cases (*Table 1*). Home inotrope was used as bridge to decision and eventual left ventricular assist device (LVAD), as bridge to stabilization, or as bridge to reverse remodelling through stimulant abstinence and guideline-directed medical therapy (GDMT).

**Table 1:** Patient characteristics at baseline and during admission

	Patient 1	Patient 2	Patient 3
DCM duration prior to admission (years)	2	17	3
Severity of DCM prior to admission	Persistently severe	2 years severe. Lost to follow-up	Persistently severe
Stimulant	Dextroamphetamine–amphetamine, methamphetamine, cocaine	Methamphetamine	Methamphetamine
Baseline echocardiogram	EF 15%, LVEDV 245 mL, mild to moderate MR	EF 15%, LVEDV 270 mL, severe MR	EF 25%, LVEDV 255 mL, severe MR
Peak lactate (mmol/L) (ref 0.5–2.2)	4.2	3.9	4.2
Peak creatinine (mg/dL) (ref 0.5–1.3)	2.6	1.7	1.8
Peak ALT (U/L) (ref <56)	290	928	169
Admission haemodynamics	BP 92/70, RA 18, PA 51/39, PCWP 38, Fick 3.5/1.6	BP 96/79, RA 10, PA 54/33, PCPW 33, Fick 2.8/1.4	BP 78/46, RA 9, PA 31/21, PCWP 20, Fick 2.0/1.1
Acute treatment	Inotrope, Impella 5.5, ProtekDuo RVAD	Inotrope	Vasopressor, inotrope, Impella 5.5, ProtekDuo RVAD
Length of stay (days)	34	16	21
Home inotrope	Milrinone	Dobutamine	Milrinone
Dose (μg/kg/min)	0.375	5.0	0.25
Home inotrope duration (months)	30	5	4
Outcome	LVAD	Reverse remodelling and inotrope wean	Stabilization and inotrope wean

BP, blood pressure; DCM, dilated cardiomyopathy; EF, ejection fraction; LVAD, left ventricular assist device; LVEDV, left ventricle end-diastolic volume; MR, mitral regurgitation; PA, pulmonary artery; PCWP, pulmonary capillary wedge pressure; RA, right atrium; RVAD, right ventricular assist device.

## Summary figure

**Figure:**
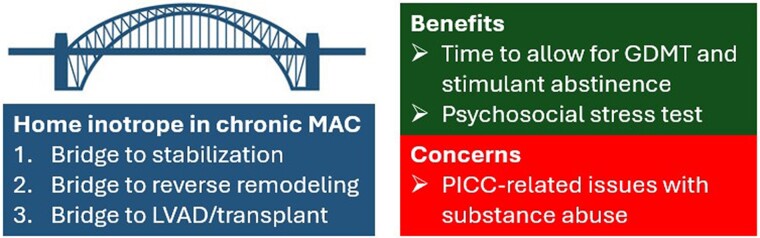


## Patient 1

A 49-year-old male had a 2-year history of severe DCM related to prescription dextroamphetamine–amphetamine and non-habitual use of methamphetamine and cocaine. He was on home milrinone since diagnosis and had been deemed ineligible for transplant for psychosocial reasons. He was now admitted with acute worsening of heart failure (HF) symptoms including dyspnoea with minimal exertion, nausea, and abdominal swelling, despite escalation of outpatient milrinone from 0.375 to 0.5 μg/kg/min. On exam, he was ill appearing, had jugular venous distension, bibasilar lung crackles, and significant leg oedema. He progressed to cardiogenic shock requiring mechanical circulatory support (MCS) with Impella 5.5 and ProtekDuo right ventricular assist device (RVAD). He was deemed eligible for LVAD by a multidisciplinary team based on his improved medical adherence in the months leading up to this admission and the fact that his condition had progressed to MCS dependence.

He underwent HeartMate 3 implantation and tricuspid valve repair (Supplementary material online, *Videos S3–S7*). He tolerated RVAD removal on post-operative Day 19. This was facilitated by improved right ventricular afterload and pulmonary artery pressures with left ventricular unloading, improved right ventricular preload by fixing severe functional tricuspid regurgitation, and weeks of peri-operative central venous decongestion. His renal function eventually normalized. He was discharged on a regimen of spironolactone 25 mg daily, bumetanide 1 mg daily, and warfarin. He is doing well 8 months after surgery, has had no LVAD complications or readmissions, is adherent to the medical regimen and office visits, and has had multiple negative toxicology screens. A transplant evaluation is currently underway.

## Patient 2

A 67-year-old male had a 17-year history of DCM related to regular methamphetamine use. His last visit with a cardiologist was 2 years prior to the current admission. At that time, his ejection fraction was severely reduced. He did not follow-up with subsequent office visits and stopped all GDMT. He was now admitted with progressively worsening HF symptoms over months including dyspnoea with minimal exertion, orthopnoea, and 7 kg weight gain. On exam, he had tachypnoea, bilateral lung crackles, and mild leg swelling. He was found to be in a low-output state with acute kidney injury and ischaemic hepatitis. He did not tolerate milrinone due to symptomatic hypotension and was switched to dobutamine. He was weaned off, but 3 days later developed worsening clinical, haemodynamic, and laboratory parameters requiring reinitiation. He had intolerance to afterload reduction. After multidisciplinary discussions, the plan was home dobutamine at the lowest dose that maintained adequate clinical and haemodynamic parameters to provide long-term stability to assess the following:

1. Adherence, methamphetamine abstinence, and workup for LVAD or transplant.
2. Initiation of GDMT and reverse remodelling.

Despite the long-standing nature of DCM, implantable cardioverter defibrillator (ICD) was deferred as he had been off GDMT for a few years. He agreed to a wearable cardioverter defibrillator (WCD).

**Figure 1:**
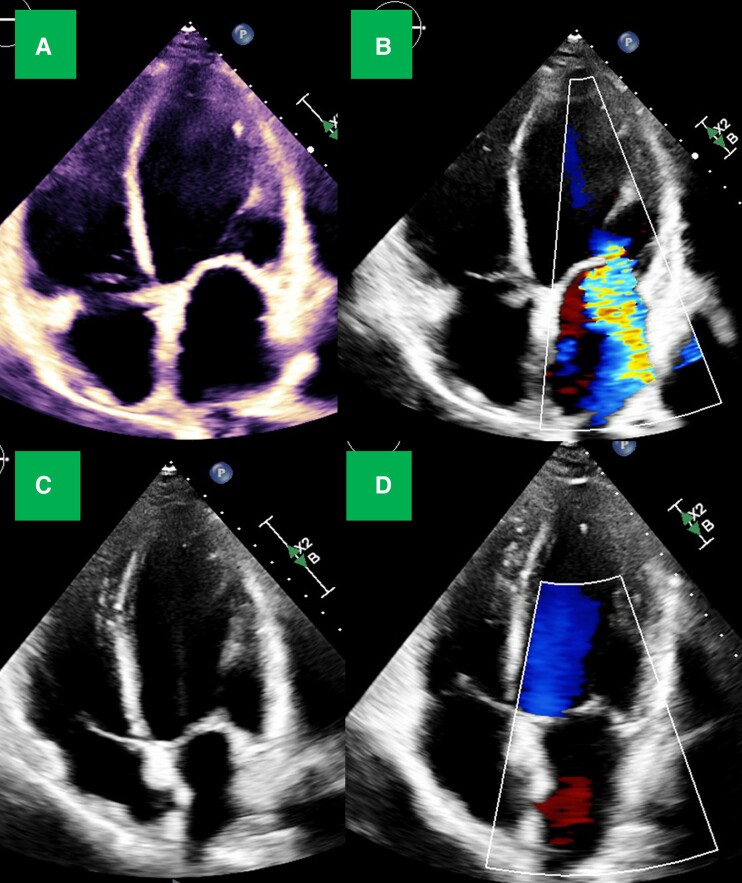
Patient 2 echocardiogram. Admission: (*A*) end-diastole, 270 mL, (*B*) systole with severe mitral regurgitation 6 months later: (*C*) end-diastole, 155 mL, (*D*) systole with no mitral regurgitation.

**Figure 2:**
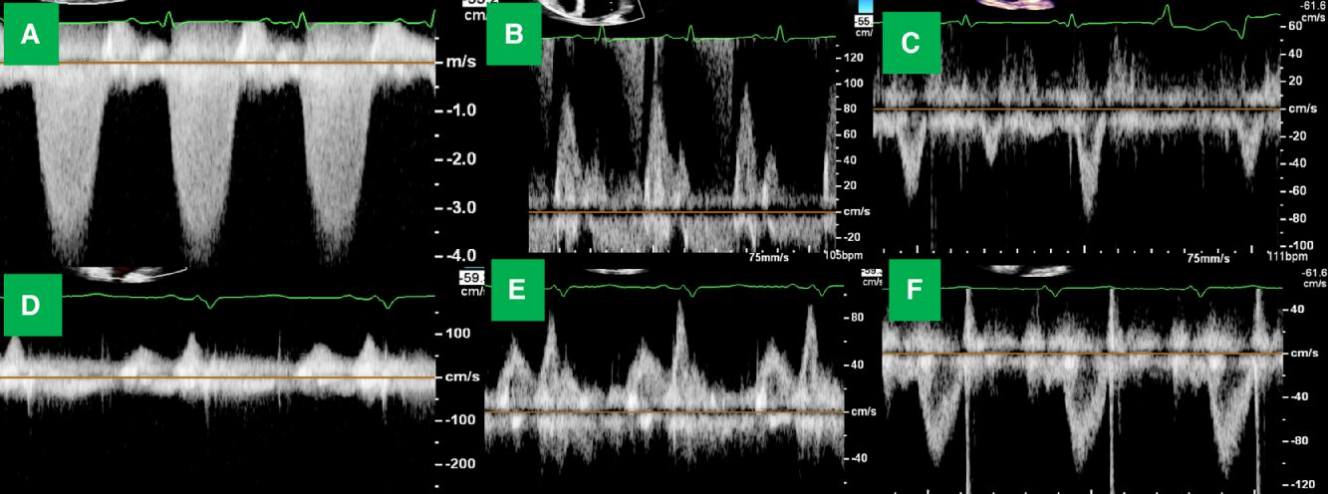
Patient 2 non-invasive haemodynamics. Admission: (*A*) mitral continuous wave Doppler showing triangular early peaking jet, (*B*) mitral inflow pattern E>>A, (*C*) severely diminished left ventricular outflow tract velocity time integral 6 months later: (*D*) absent mitral continuous wave Doppler signal, (*E*) mitral inflow with E/A reversal and relatively small E-wave, (*F*) normal left ventricular outflow tract velocity time integral.

Following discharge, he demonstrated excellent adherence to office visits and use of WCD, averaging ∼17 h/day on device interrogation. Functional capacity significantly improved. Despite inability to add beta-blocker, his resting heart rate improved to 65–75 b.p.m. in the setting of methamphetamine abstinence. Blood pressure improved allowing for initiation of GDMT. Given dramatic improvement, he was electively admitted 5 months later for haemodynamic-guided inotrope wean, which he tolerated. After 8 months, he was tolerating quadruple therapy with metoprolol succinate 25 mg daily, sacubitril–valsartan 24–26 mg twice daily, spironolactone 25 mg daily, and dapagliflozin 10 mg daily. He is New York Heart Association (NYHA) class 1, B-type natriuretic peptide < 100 pg/mL, and has evidence of reverse remodelling. His left ventricular size improved from moderately dilated to normal, ejection fraction improved from 15 to 40%, and severe mitral regurgitation resolved (*Figures 1* and *2*, Supplementary material online, *Videos S1* and *S2*).

## Patient 3

A 34-year-old male had a 3-year history of severe DCM secondary to regular methamphetamine use. He had been NYHA class 1–2 but was now admitted with acute HF symptoms including dyspnoea at rest. On exam, he was in respiratory distress with significant bilateral lung crackles, cool extremities, and mild skin mottling of his feet. He was in cardiogenic shock and new-onset atrial fibrillation with rate of 170 b.p.m. He underwent emergent cardioversion but remained unstable, requiring MCS with Impella 5.5 and later required ProtekDuo RVAD. After improvement of haemodynamic and laboratory parameters, he was weaned off MCS to milrinone with a total of 13 days Impella support and 10 days RVAD support. He failed attempts to wean milrinone despite afterload reduction. After multidisciplinary discussions, the plan was home milrinone with a similar rationale to Patient 2. Considering he had been on GDMT for 3 years and was being discharged on milrinone, he agreed to ICD. Despite his advanced HF status, candidacy for advanced therapies had not been excluded, and there remained a possibility of long-term myocardial stabilization with methamphetamine abstinence and preventing tachyarrhythmia.

Prior to admission, he was intolerant of beta-blockers developing low-output symptoms including nausea. His heart rate had consistently been sinus 100–120 b.p.m. during office visits. After discharge on milrinone and with methamphetamine abstinence, he eventually tolerated quadruple therapy including high-dose beta-blocker. Metoprolol was slowly titrated to 150 mg daily with resting heart rate 70–80 b.p.m. Other medications included losartan 50 mg twice daily, spironolactone 25 mg daily, and empagliflozin 10 mg daily. The patient was concerned about the psychosocial aspects of LVAD and transplant and felt a burden even with the peripherally inserted central catheter (PICC). Given significant improvement, it was decided to proceed with haemodynamic-guided inotrope wean. He opted to avoid admission and was successfully weaned based on clinical parameters. Four months following milrinone wean, he is NYHA class 1–2, but echocardiogram has yet to demonstrate improvement. Since milrinone wean, adherence with follow-up has been a challenge.

## Discussion

Catecholaminergic stimulants include medications such as methylphenidate and dextroamphetamine–amphetamine as well as illicit drugs such as methamphetamine and cocaine. Methamphetamine displaces neurotransmitters into the synapse and inhibits their reuptake. Compared to amphetamine, methamphetamine possesses an extra methyl group contributing to higher lipophilic activity, which enhances central nervous system (CNS) activity and duration of action. Their potential to cause cardiomyopathy is related to not only indirect adrenergic stimulation but also direct myocardial injury through oxidative stress, apoptosis, altered metabolism, altered gene expression, and abnormal intracellular calcium homeostasis.^2^ A recent study based on a national database in the USA showed a significant rise in HF hospitalizations related to methamphetamine-associated cardiomyopathy (MAC), with a higher proportional increase among middle-aged men,^3^ although when compared to other causes of cardiomyopathy, patients with MAC are generally younger.^1^ The epidemiology and cardiac manifestations were also highlighted in national studies from Australia and Germany.^4,5^

Addiction to stimulants poses a unique challenge in the setting of severe DCM. In addition to the inherently addictive potential of these substances due to CNS effect, their inotropic effect can palliate debilitating low-output symptoms. This phenomenon was mentioned by Patient 2 when describing the challenges he faced abstaining from methamphetamine. The indirect positive inotropic action of methamphetamine has been demonstrated in a human atrial model and animal studies.^6,7^

Although acute management of MAC has been described in case series,^1^ including a subset requiring short-term inotrope or MCS, the use of home inotrope as a bridge to recovery or advanced therapies in those with chronic MAC has not been well described.

The potential for reverse remodelling in chronic MAC is unclear. Reassuringly in animal studies, histopathologic cardiac abnormalities induced by daily high-dose injection of methamphetamine for 12 weeks demonstrated pronounced recovery after 8–12 weeks of withdrawal.^8^ In humans, long-term methamphetamine use was associated with a lower chance of reverse remodelling if fibrosis was present on cardiac magnetic resonance imaging.^9^ In our series, Patient 2 had significant reverse remodelling after drug abstinence and quadruple therapy despite the long-standing nature of his MAC. In those that fail to improve, LVAD and transplant are options. However, LVAD with active substance abuse is associated with negative outcomes.^10^

Home milrinone was favoured in our patients, although Patient 2 did not tolerate milrinone due to his initial vasodilatory state. Beyond the phase of acute stabilization, beta-blockers can be combined with home milrinone but not dobutamine due to their competing mechanism of action. This combination was associated with improved survival in two large studies of contemporary home inotropic therapy.^11,12^ It should be noted that the historic trials that demonstrated increased mortality with home inotrope were in an era prior to beta-blockers and defibrillators, and an era when inotropes were combined with an additional calcitrope in the form of digoxin, which was historically used at higher concentrations.^13^

An important dilemma exists regarding PICC in those with substance abuse. A literature review for outpatient parenteral antimicrobial therapy in this high-risk population revealed reassuring data on safety, efficacy, mortality, catheter-related adverse events, and misuse of venous catheter.^14^ Limitations include retrospective small studies and shorter-term therapy with duration ranging from 18 to 42 days. Patients in our series had no PICC-related issues with a duration ranging from 4 to 30 months accounting for a selection bias. Medical adherence improved. In Patients 1 and 2, this adherence was sustained for >1–3 years, but in Patient 3, there were setbacks after 4 months. In the inpatient setting, patients and family members are provided with education on managing the PICC and pump. Following discharge, they initially have visits from the home health nurse two to three times weekly with the goal of achieving independence. Ability to handle PICC dressing changes, medication bag changes, and pump troubleshooting provides valuable psychosocial information when evaluating for advanced therapies. Interrogation of WCD provides similar information. Patients with stimulant-induced DCM are not a homogenous group. They have a wide psychosocial spectrum related to social support, housing and job situations, adherence, and level of insight. When assessing their candidacy for home inotrope and advanced therapies, a nuanced and personalized approach should be adopted.

In conclusion, in patients with chronic stimulant-induced DCM admitted with cardiogenic shock, we believe home inotrope can be utilized in well-selected cases through a multidisciplinary ‘heart team’ approach. In this setting, home inotrope can provide long-term stabilization to assess psychosocial candidacy for advanced therapies, can serve as a ‘psychosocial stress test’, and in a subset of patients can allow time for reverse remodelling through methamphetamine abstinence and titration of GDMT.

## Lead author biography


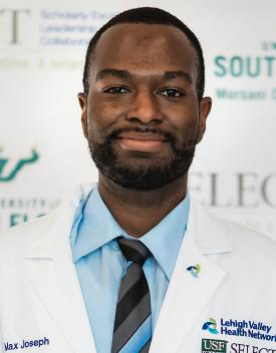


I am currently a third-year medical student completing my core clerkship rotations. The experiences have given me better insight into the intricacies of medicine while building my knowledge base. Additionally, I am recognizing what my passions are and which path in medicine is best for me. I have been fascinated by cardiology and the complexities of cardiovascular disease processes. My future interests therefore are in internal medicine and cardiology with great interest in preventative care.

## Supplementary Material

ytae406_Supplementary_Data

## Data Availability

No new data were generated or analysed in support of this research. All data are present within the manuscript.

## Notes

**Consent:** Consent has been obtained from patients per COPE guidelines.

**Funding:** None declared.

## References

[1] Reddy PKV , Ng TMH, Oh EE, Moady G, Elkayam U. Clinical characteristics and management of methamphetamine-associated cardiomyopathy: state-of-the-art review. J Am Heart Assoc 2020;9:e016704. 32468897 10.1161/JAHA.120.016704 PMC7428977

[2] Jafari Giv M . Exposure to amphetamines leads to development of amphetamine type stimulants associated cardiomyopathy (ATSAC). Cardiovasc Toxicol 2016;17:13–24. 10.1007/s12012-016-9385-8 27663745

[3] Al-Yafeai Z , Ali S, Brown J, Venkataraj M, Bhuiyan MDS, Faisal ASM, et al Cardiomyopathy-associated hospital admissions among methamphetamine users. JACC Adv 2024;3:100840. 39130045 10.1016/j.jacadv.2024.100840 PMC11312042

[4] Darke S , Duflou J, Kaye S. Prevalence and nature of cardiovascular disease in methamphetamine-related death: a national study. Drug Alcohol Depend 2017;179:174–179. 28787694 10.1016/j.drugalcdep.2017.07.001

[5] Schwarzbach V , Lenk K, Laufs U. Methamphetamine-related cardiovascular diseases. ESC Heart Fail 2020;7:407–414. 31950731 10.1002/ehf2.12572 PMC7160483

[6] Neumann J , Hußler W, Azatsian K, Hofmann B, Gergs U. Methamphetamine increases force of contraction in isolated human atrial preparations through the release of noradrenaline. Toxicol Lett 2023;383:112–120. 37394154 10.1016/j.toxlet.2023.06.012

[7] Ishiguro Y , Morgan JP. Biphasic inotropic effects of methamphetamine and methylphenidate on ferret papillary muscles. J Cardiovasc Pharmacol 1997;30:744–749. 9436813 10.1097/00005344-199712000-00008

[8] Islam MN , Jesmine K, Kong Sn Molh A, Hasnan J. Histopathological studies of cardiac lesions after long term administration of methamphetamine in high dosage—part II. Leg Med (Tokyo) 2009;11:S147–S150. 19345131 10.1016/j.legalmed.2009.02.035

[9] Pujol-López M , Ortega-Paz L, Flores-Umanzor EJ, Perea RJ, Bosch X. Cardiac magnetic resonance as an alternative to endomyocardial biopsy to predict recoverability of left ventricular function in methamphetamine-associated cardiomyopathy. JACC Heart Fail 2017;5:853–854. 29096799 10.1016/j.jchf.2017.08.009

[10] Cogswell R , Smith E, Hamel A, Bauman L, Herr A, Duval S, et al Substance abuse at the time of left ventricular assist device implantation is associated with increased mortality. J Heart Lung Transplant 2014;33:1048–1055. 25107352 10.1016/j.healun.2014.06.009

[11] Sami F , Acharya P, Noonan G, Maurides S, Al-Masry AA, Bajwa S, et al Palliative inotropes in advanced heart failure: comparing outcomes between milrinone and dobutamine. J Card Fail 2022;28:1683–1691. 36122816 10.1016/j.cardfail.2022.08.007

[12] Zaghlol R , Ghazzal A, Radwan S, Zaghlol L, Hamad A, Chou J, et al Beta-blockers and ambulatory inotropic therapy. J Card Fail 2022;28:1309–1317. 35447337 10.1016/j.cardfail.2022.03.352

[13] Ahmad T , Miller PE, McCullough M, Desai NR, Riello R, Psotka M, et al Why has positive inotropy failed in chronic heart failure? Lessons from prior inotrope trials. Eur J Heart Fail 2019;21:1064–1078. 31407860 10.1002/ejhf.1557 PMC6774302

[14] Suzuki J , Johnson J, Montgomery M, Hayden M, Price C. Outpatient parenteral antimicrobial therapy among people who inject drugs: a review of the literature. Open Forum Infect Dis 2018;5:ofy194. 30211247 10.1093/ofid/ofy194 PMC6127783

